# Implementation Measures of the obligation of care (according to article 30 in Tunisian law 92-83 of the year 1992) at the psychiatric department of Monastir

**DOI:** 10.1192/j.eurpsy.2023.1863

**Published:** 2023-07-19

**Authors:** B. Amamou, M. Ben Mbarek, I. Betbout, A. Jabeur, F. Zaafrane, L. Gaha

**Affiliations:** 1 Psychiatry, Faculty of Medicine of Monastir University of Monastir; 2 Departement of Psychiatry, EPS fattouma Bourguiba Monastir, Monastir, Tunisia

## Abstract

**Introduction:**

Consent to care remains the general principle and care without consent must be the exception. However, resorting to care without consent in psychiatry may be indicated when awareness of the disorders or recognition of the need for care is impaired. For this purpose, we have focused on the questions concerning the degree of applicability of this procedure of obligation of care in Tunisia.

**Objectives:**

The purpose of this study is to describe implementation measures of the obligation of care among patients in the psychiatric department of Monastir.

**Methods:**

This is a retrospective, descriptive and qualitative study of five patients followed in the psychiatric department at the Fattouma Bourguiba University Hospital of Monastir between January 2020 and August 2022 who were subjects to the obligation of care with reference to Article 30 of Law 92-83 of August 3, 1992, amended by Law No. 2004-40 of May 3, 2004.

**Results:**

All patients in the study were males. Two out of five patients had a higher level of education. Two patients had a history of family psychiatric disorders. Two patients were being followed for schizophrenia, two others had schizoaffective disorder and one patient had a chronic delusional disorder. Only one patient had a duration of untreated psychosis of 6 years. Most of the patients had been hospitalized once to four times prior to their submission to article 30 of the obligation of care. Only one patient was hospitalized at the request of a third party, four patients were hospitalized by order of the court. Four out of five patients were treated with antipsychotic drugs (delayed-release form). After their submission to article 30, three patients were readmitted for decompensation due to treatment discontinuation, one patient did not consult the doctors, and only one of the five patients was present with his family at his post-treatment appointment.

**Conclusions:**

what we deduced from our observation encourages us to work better on improving laws and training dedicated to psychiatric practitioners to optimize this judicial and therapeutic practice in our country.

**Disclosure of Interest:**

None Declared

